# Targeting tauopathy with engineered tau-degrading intrabodies

**DOI:** 10.1186/s13024-019-0340-6

**Published:** 2019-10-22

**Authors:** Gilbert Gallardo, Connie H. Wong, Sara M. Ricardez, Carolyn N. Mann, Kent H. Lin, Cheryl E. G. Leyns, Hong Jiang, David M. Holtzman

**Affiliations:** 1 Department of Neurology, Washington University School of Medicine, St. Louis, MO 63110 USA; 2 Hope Center for Neurological Disorders, Washington University, Campus Box 8111, 660 S. Euclid Avenue, St. Louis, MO 63110 USA; 3 Charles F. and Joanne Knight Alzheimer’s Disease Research Center, Washington University, St. Louis, MO USA; 4 Department of Molecular Genetics and Center for Translational Neurodegeneration Research, University of Texas Southwestern Medical Center, Dallas, TX 75390 USA; 5 Neuroscience Discovery, Merck Research Laboratories, Boston, MA 02115 USA

**Keywords:** Alzheimer’s disease, Tauopathy, Immunotherapy, Intrabodies, Tau degradation

## Abstract

**Background:**

The accumulation of pathological tau is the main component of neurofibrillary tangles and other tau aggregates in several neurodegenerative diseases, referred to as tauopathies. Recently, immunotherapeutic approaches targeting tau have been demonstrated to be beneficial in decreasing tauopathy in animal models. We previously found that passive immunotherapy with anti-tau antibody to human tau or expression of an anti-tau secreted single-chain variable fragment (scFv) in the central nervous system of a mouse model of tauopathy decreased but did not remove all tau-associated pathology. Although these and other studies demonstrate that conventional immunotherapeutic approaches targeting tau can influence tau pathogenesis, the majority of pathological tau remains in the cytosol of cells, not typically accessible to an extracellular antibody. Therefore, we reasoned targeting intracellular tau might be more efficacious in preventing or decreasing tauopathy.

**Methods:**

By utilizing our anti-tau scFv, we generated anti-tau intrabodies for the expression in the cytosol of neurons. To enhance the degradation capacity of conventional intrabodies, we engineered chimeric anti-tau intrabodies fused to ubiquitin harboring distinct mutations that shuttle intracellular tau for either the proteasome or lysosomal mediated degradation. To evaluate the efficacy in delaying or eliminating tauopathy, we expressed our tau degrading intrabodies or controls in human tau transgenic mice by adeno-associated virus prior to overt tau pathology and after tau deposition.

**Results:**

Our results demonstrate, the expression of chimeric anti-tau intrabodies significantly reduce tau protein levels in primary neuronal cultures expression human tau relative to a non-modified anti-tau intrabody. We found the expression of engineered tau-degrading intrabodies destined for proteasomal-mediated degradation are more effective in delaying or eliminating tauopathy than a conventional intrabody in aged human tau transgenic mice.

**Conclusion:**

This study, harnesses the strength of intrabodies that are amendable for targeting specific domains or modifications with the cell-intrinsic mechanisms that regulate protein degradation providing a new immunotherapeutic approach with potentially improved efficacy.

## Background

Tau was initially identified as a microtubule-associated protein that subsequently was found to be the main component of neurofibrillary tangles and other aggregated forms of tau in several neurodegenerative diseases, referred to as tauopathies [1–4]. These diseases include several neurodegenerative disorders such as progressive supranuclear palsy, corticobasal degeneration, Pick’s disease, and certain forms of frontotemporal dementia. Alzheimer’s disease, the most common cause of dementia, is a secondary tauopathy that is accompanied by the presence of amyloid-β pathology. Within the central nervous system (CNS), tau is predominately expressed in neurons and maintained at higher concentrations within axons to promote microtubule assembly and stability [5]. The regulation of tau binding to microtubules is mediated by the phosphorylation of serine and threonine residues at sites immediately adjacent to or within the microtubule-binding domain (MBD) [2]. Phosphorylation within or around the MBD alters the conformation of the MBD decreasing its affinity for microtubule binding and liberating tau [6] . The accumulation of hyperphosphorylated, aggregated tau is present in insoluble paired helical filaments and other structures that result in tau inclusions.

Emerging evidence now suggests the trans-cellular propagation of tau pathology mediates the progression of tauopathy in these diseases [7–9]. Therefore, immunotherapeutic approaches that target extracellular tau could be a way to decrease the spread of tau pathology. In line with this notion, recent studies have demonstrated immunotherapy with different anti-tau antibodies prevented the accumulation of pathological tau in mouse models of tauopathies^10–17^. Active vaccination studies in tau transgenic mice targeting aberrant phosphorylated tau protein have also been shown to reduce tau-associate pathology and improve behavioral abnormalities [10, 11]. Similar to active immunotherapies, passive immunotherapy before or soon after the onset of tauopathy with anti-tau monoclonal antibodies (mABs) also provided some beneficial effects in transgenic tau mice [12–16].

In former studies, we developed and assessed an anti-tau mAB (HJ8.5) that blocked cellular tau seeding in a biosensor assay [17–19]. In addition, passive immunization with the HJ8.5 anti-tau mAB in transgenic mice expressing human P301S tau (P301S-tg) decreased tau-associated pathology, slowed disease progression, and improved cognitive deficits [17–19]. In additional immunotherapy studies, we also generated anti-tau single-chain variable fragments (scFvs) to evaluate the necessity of the Fc domain, as a potential safety concern in general for immunotherapies is the development of adverse effects due to an inflammatory response by IgG activation of FcγRs on microglia [20, 21]. The expression of the secreted anti-tau scFvs by adeno-associated virus (AAV) – mediated gene transfer into the CNS led to a significant decrease in the accumulation of pathological tau in aged P301S-tg mice, likely by sequestering extracellular tau [20]. These studies validate the efficacy of anti-tau scFvs and suggest that Fc-dependent microglial mediated tau clearance is likely not mandatory for anti-tau antibodies to elicit neuroprotective effects. Although these studies support immunotherapeutic approaches with either a secreted anti-tau scFv or a conventional antibody being sufficient to delay tau pathogenesis, the majority of pathological tau remains intracellular in the cytosol, making that location of tau likely inaccessible to both cellularly secreted scFvs and full-length antibodies administered intraperitoneally, subcutaneously, intravenously, or intracerebroventricularly. Thus, targeting intracellular tau with anti-tau scFvs expressed intracellularly in the cytosol (intrabodies) may be more efficacious in preventing tauopathy progression and even in removing existing pathological forms of intracellular tau.

Advances in recombinant antibody technology have provided the ability to express intrabodies intracellularly in the cytosol that function by impairing protein-protein interactions or neutralizing protein function based on the epitope targeted. However, the neutralizing mechanisms of conventional intrabodies limit their efficacy when targeting pathological proteins such as tau, in which protein interactors that are detrimental remain unknown and identifying intrabodies that neutralize the aggregation of intracellular tau is somewhat challenging. To overcome this potential obstacle and limitation, we have engineered chimeric anti-tau intrabodies fused to ubiquitin harboring distinct mutations that make the intrabody prone for either proteasomal or lysosomal degradation with the goal of targeting intracellular tau for degradation. We hypothesized that expression of the modified tau-degrading intrabodies in neurons would reduce intracellular tau protein levels leading to a greater neuroprotective effect relative to conventional immunotherapeutic approaches for tauopathies.

In this study, we show the expression of chimeric anti-tau intrabodies significantly reduces tau protein levels in primary neuronal cultures expressing P301S human tau (h-tau) relative to the conventional anti-tau intrabody containing no modifications. By utilizing AAV2/9-mediated gene transfer, we expressed our anti-tau intrabodies or controls in P301S-tg mice prior to overt tau pathology. Intriguingly, despite the ability for both chimerically fused anti-tau intrabodies to decrease P301S h-tau in primary neurons, the expression of anti-tau intrabodies selective for proteasomal mediated degradation in aged P301S-tg mice displayed effects on preventing the accumulation of pathological tau. In contrast, expression of the anti-tau intrabody that targets lysosomal-mediated degradation or the conventional anti-tau intrabody were both ineffective in preventing tauopathy. Additionally, we expressed our anti-tau intrabodies or controls in aged P301S-tg mice after the onset of tauopathy by AAV-mediated gene transfer. Similar to the effects we observed with the expression of anti-tau intrabodies prior to overt tauopathy, expressing the anti-tau intrabody selective for proteasomal mediated degradation displayed effects on decreasing pathological tau in aged P301S-tg mice.

## Methods

### Animal model of Tauopathy

The mouse model utilized for the present study were transgenic mice expressing human P301S tau (P301S-tg) purchased from The Jackson Laboratory and bred on a B6C3 background^16^. P301S mice develop widespread tau-associated pathology that is age dependent primarily in the hippocampus, parts of the neocortex, amygdala, piriform cortex, and entorhinal cortex. In this study, male age matched P301S mice littermates were analyzed due to the high variability in tau-associated pathology in aged P301S female mice [14, 15]. All experiments were conducted under the institutional guidelines and were approved by the Institutional Animal Care and Use Committee at Washington University School of Medicine. At the time of endpoint, mice were anaesthetized before cardiac perfusion with PBS/heparin followed by brain dissection and immersion in 4%PFA. Following 48 h, the PFA was exchanged with 30% sucrose and the brain was incubated for an additional 48 h before freezing with 2-Methylbutane on dry ice and stored at − 80 °C before sectioned on a microtome (30 μm thickness). Floating sections were kept in 24-well plates with 30% ethylene glycol, 15% sucrose and 0.2 M sodium phosphate at − 20 °C until used.

### AAV injections

All intrabodies were cloned into the AAV vector under the control of the chicken-β-actin (CBA) promoter. All AAV used in these studies were produced at the *Hope Center Viral Vectors Core at Washington University School of Medicine*. Eight month-old P301S mice were anesthetized with isofluorane (4% induction and 2.0% maintenance) followed by bupivacaine (2 mg/kg) administration subcutaneously prior to the incision on the scalp. Once anesthetized, the hair above the skull was shaved and swabbed with Betadine for sterilization. The animal was then placed onto a small animal Kopf stereotaxic apparatus equipped with an anesthetic mask. The skin was resected just posterior to the eyes to the base of the skull and the skull is cleaned with sterile artificial cerebrospinal fluid. The virus was injected unilaterally into the hippocampus at coordinates bregma: − 2.5 mm posterior, 2.0 mm horizontal from midline (right), at a depth of 2.2 mm utilizing a Hamilton syringe (1702RNR) at 0.5 μl/minute for a final volume of 2.5 μl. The Hamilton syringe was then left for an additional five minutes and gently withdrawn.

### Characterization of intrabodies in 293 t (HEK cells)

Prior to plating HEK cells, plates were coated with 10 μg ml^− 1^ poly-L-lysine (PLL, Sigma, P2636) 10 min followed by brief washes with water. The HEK293t cells were plated in DMEM supplemented with 10% FBS at a density of 60 K cells/well. Following 24 h HEK293t cells were transiently transfected with Lipofectamine according to the guidelines of the manufacturer (ThermoFisher). The concentrations of transfected intrabody expressing plasmids ranged from 0.1μg, 0.5μg, 1.0 μg, and 2.0μg pDNA together with co-transfection of 0.2μg pDNA of a tau expressing plasmid. Following 72 h post-transfection the HEK293t whole cell lysates were collected in standard lysis buffer (50 mM Tris-buffer, 150 mM NaCl, 1 mM EDTA, 1% triton and a cocktail of protease and phosphatase inhibitors) and 5μg of total protein lysate was subjected to SDS-PAGE immunoblotting analysis. For proteasomal inhibition, at 72 h post-transfection the cells were treated with 50 μm MG132 (Sigma Aldrich M7449) for 90 min followed by collection in standard lysis buffer. For lysosomal inhibition, at 72 h post-transfection the cells were treated with 500 μm bafilomycin (Sigma Aldrich B1793) for 6 h followed by collection in standard lysis buffer. Afterwards, to detect both the expression of each intrabody, controls, and the relative tau protein levels, immunoblots were subjected to anti-HA conjugated HRP (clone 3F10, Roche; 1:10,000) and human tau-specific antibody anti-HJ8.5 previously characterized^14^.

### Primary neuronal cultures expressing human tau and the intrabodies

We performed neuronal cultures from E17 wild-type mice, described previously^27^. For immunofluorescence analysis of the relative levels of human tau protein, primary cortical neurons were infected with AAV2/8-synapsin-P301S tau virus for 6 h on ice. Cells were then pelleted, washed, and plated onto 24-well tissue culture plates glass cover slips pre-coated with 10 μg ml^− 1^ poly-L-lysine (PLL, Sigma, P2636) at a density of 150,000 cells per well in neurobasal medium. Following 72 h post-transduction with AAV-human P301S tau, the primary neuronal cultures were infected with AAV2/9-CBA encoding the various anti-tau intrabodies and cultured for an additional seven days. For analysis, primary neuronal cultures were fixed in 4% PFA, 4% sucrose for 10 min followed by permeabilization with 0.3% triton in PBS for 10 min and stained with human specific tau antibody Ht7B (Thermo Fisher, MN1000B).

For measurements of the relative human P301S tau protein in primary neuronal cultures following the expression of the various anti-tau intrabodies, primary cortical neurons were infected with AAV2/8-synapsin-P301S tau virus for 6 h on ice. Cells were then pelleted, washed, and plated onto 6-well tissue culture plates pre-coated with 10 μg ml^− 1^ poly-L-lysine (PLL, Sigma, P2636) at a density of 400,000 cells per well in neurobasal medium. Following 72 h post-transduction with AAV-human P301S tau, the primary neuronal cultures were infected with AAV2/9-CBA encoding the various anti-tau intrabodies and cultured for an additional seven days. For analysis, 200 μl of standard RIPA buffer was added to each well for collection of total neuronal protein lysates followed by sonication. For human tau-specific measurements in neuronal lysates, a Sandwich-ELISA was used. Plates were coated with Tau5 (gift from L. Binder, Northwestern University, Chicago, Illinois, USA) and blocked with 4% BSA in PBS. Samples were diluted in sample buffer and as a detector antibody biotinylated anti-htau (clone HT7, Thermo Fisher Scientific MN1000B) followed by streptavidin-HRP (65R-S104PHRP; Fitzgerald) was used. All ELISAs were developed with super slow TMB substrate solution (Sigma) before reading on a plate reader at 650 nm.

### Immunohistochemistry

Utilizing the free-floating method, we subjected unilaterally injected AAVs encoding intrabody^-K48R^, intrabody^-K63^, conventional intrabody, or AAV-control injected mice brain sections to pathological tau marker anti-AT8 (phospho-tau Ser202, Thr205, Thermo Scientific, MN1020B) and for detection of the various intrabodies anti-HA (Vector Laboratories and Bethyl Laboratories, Inc.). Brain sections (30 μm) were picked that displayed a clear structured cross-section of the hippocampus, piriform, and entorhinal cortex regions. For quenching auto fluorescence, the sections were incubatated in sudan black (40 mg in 40 ml of 70% EtOH) for 10 min followed by washing with 0.03% PBS-Tween. Nonspecific binding was blocked by PBS 3% BSA, 3% normal goat serum 0.1% Triton X-100. Brain sections were then incubated with primary antibodies, rabbit anti-HA (1:1000), and anti-AT8 (1:500) overnight at 4C. Thereafter, the sections were washed three times in PBS for 10 min each followed by incubation with anti-streptavidin conjugated 568 (Molecular Probes 1:500), and anti-rabbit conjugated 488 (Molecular Probes 1:500) for 2 h at room temperature in the dark. Sections were then washed three times in PBS for 20 min each and mounted with ProLong Gold anti-fade reagent. Images were taken with an epi-fluorescence microscope at 4× magnification and quantified using MetaMorph as previously reported^27^. For analysis of p-tau (anti-AT8), the dentate gyrus, granule cell layer, mossy fibers, CA1, CA2, CA3 regions were traced from a minimum of 2 sections (300 μm) apart from each other per mouse using MetaMorph. To determine the percent area covered per region, we quantified the average ratio of p-tau (AT8) positive area from the contralateral to ipsilateral side.

To measure total tau levels brain sections from mice injected unilaterally with AAVs encoding intrabody^-K48R^, intrabody^-K63^, conventional intrabody, or AAV-control injected were subjected to immunohistochemistry with human anti-HT7 (Thermo Fischer, MN1000B). Free floating whole brain sections cut at 30 μm were incubated in .3% H_2_O_2_ in TBS for 10 min at room temperature to quench endogenous peroxidase. Sections were blocked in 3% milk .25% Triton X-100. Sections were incubated with primary antibody anti-HT7(1:500) for 1 h at room temperature. ABC Elite solution (1:400) was prepared from VECTASTAIN Elite ABC system (Vector Labs) and sections were incubated in solution for 1 h at room temperature. Staining was developed for 5 min with DAB plus .01% Nickel and .0005% H_2_O_2._ Sections were then dipped in ethanol and Xylene to clear any excess DAB and mounted with Cytoseal. Images were captured using the Olympus Nanozoomer 2.0-HT (Hammatsu). Images were then exported to ImageJ and for analysis of total tau, the dentate gyrus, granule cell layer, mossy fibers, Ca1, Ca2, Ca3, striatum radiatum, and hippocampal fissure were manually traced from 2 sections per mouse. To determine the percent area covered per region, we quantified the average ratio of tau positive area from contralateral to ipsilateral side.

### Fluorescence in-situ hybridization

For generation of the RNA probe, the anti-tau scFV plasmid was cloned into the pCR II-TOPO vector using the TOPO™ TA Cloning™ Kit, Dual Promoter (ThermoFisher 450,640). The plasmid was then digested with Not1 for 1 h and subsequently purified. The linearized DNA was then transcribed into RNA in the presence of DIG-UTP using the DIG RNA Labeling Kit (SP6/T7) using standard protocol procedures (Sigma-Aldrich (11,175,025,910 Roche).

Mounted sections were incubated with 1 mg/mL pepsin in 0.2 M HCl at 37 °C for 7 min for antigen retrieval followed by three washes of 5 min in PBS. The sections were then incubated twice in 0.1 M phosphate buffer (PB) for 10 min, twice in 0.75% glycine in 0.1 M PB for 15 min, and once in 0.3% Triton X-100 in 0.1 M PB for 20 min, followed by a 5 min wash in PB. For cell permeabilization the sections were incubated for 30 min at 37 °C in 1 M Tris-Cl 0.5 M EDTA buffer containing 0.5 μg/mL proteinase K followed by a single wash in 0.75% glycine for 5 min and one wash in 5X SSC for 5 min. Following the proteinase K digestion, the sections were incubated in hybridization buffer (5X SSC buffer containing 50% formamide, 1x Denhardts, 10 mg/ml salmon sperm DNA, 12.5 mg *E. coli* tRNA) for 2 h at room temperature. The RNA probe was diluted (1uL/100uL) in hybridization buffer, heated at 80 °C for 5 min prior to applying to the sections which were placed in a vertical chamber humidified with 5X SSC in 50% formamide overnight at 65 °C. The next day, the slides were submerged in pre-warmed 5X SSC for 5 min at 65 °C followed by 3 washes in 0.2% SSC for 30 min each at 65 °C. Sections were then blocked with 0.25% PBS-Triton X-100 5% normal goat serum for 30 min at room temperature followed by incubating with the anti-phospho-tau mAB (AT8 1:500) in 3% BSA-PBS .1% triton overnight at 4 °C. Following three consecutive washes in PBS for 10 min. Fluorescently labeled secondary antibodies were diluted 1:500 in 3% BSA-PBS and applied to the sections for 2 h at room temperature. After three 20 min washes with PBS, sections were coverslipped with Prolong Gold with DAPI (Invitrogen).

### Statistical analysis

Blinding and randomization was performed on all analysis. All graphs represent means ± SEM. Statistical analysis was performed with GraphPad Prism 5.01 using one-way ANOVA with Tukey’s multiple comparison.

## Results

### Engineering anti-tau intrabodies designed for proteasome or lysosomal tau-mediated degradation

**Fig. 1:**
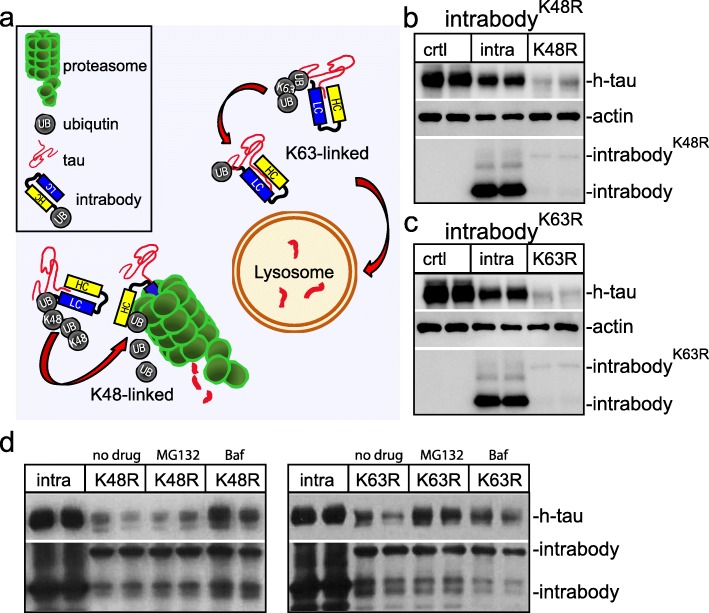
Engineering chimeric tau-degrading intrabodies fused to ubiquitin for proteasome or lysosomal-mediated degradation. **a** Diagram depicts that K48-linked polyubiquitin tau-intrabodies shuttle tau for proteasome degradation, whereas, K63-linked polyubiquitin tau-intrabodies shuttle tau for lysosomal degradation. **b** Immunoblotting analysis of HEK293t cell lysates co-expressing h-tau together with either a conventional anti-tau intrabody or the anti-tau intrabody^K48R^ revealed the chimeric anti-tau intrabody fused to ubiquitin harboring a K48R mutation decreased tau protein levels relative to the conventional intrabody or control. Protein levels are relative to actin. The expression of the anti-tau intrabody^K48R^ also revealed a lower band that potentially corresponds to a cleaved form of the chimeric intrabody. **c** Immunoblotting analysis of HEK293t cell lysates co-expressing h-tau together with either a conventional anti-tau intrabody or the anti-tau intrabody^K63R^ revealed the chimeric anti-tau intrabody fused to ubiquitin harboring a K63R mutation decreased tau protein levels relative to the conventional intrabody and control. Similarly, expression of the anti-tau intrabody^K63R^ also revealed a lower band that potentially corresponds to a cleaved form of the chimeric intrabody. **d** Immunoblotting analyses of HEK293t cell lysates co-expressing h-tau together with either a conventional anti-tau intrabody, anti-tau intrabody^K63R^ or anti-tau intrabody^K48R^ following proteasomal (MG132) or lysosomal (Baf) inhibition

We first set out to determine if shuttling our anti-tau intrabody, derived from anti-tau antibody HJ8.5, for either proteasomal or lysosomal degradation pathways by the ubiquitin system substantially decreases intracellular tau protein levels. Ubiquitin is a highly conserved small regulatory protein that is covalently attached to lysine residues of target proteins. The ubiquitination of target proteins may regulate either their cellular localization, protein interactions, or degradation that is dependent on either monoubiquitination or polyubiquitination. The polyubiquitination occurs at one of seven lysine residues of ubiquitin, most predominantly at the lysine-48 (K48) or lysine-63 (K63). A K48-linked polyubiquitin chain targets proteins for destruction by shuttling them to the 26S proteasome (Fig. 1a) [22]. In contrast, K63-linked polyubiquitination induces protein degradation predominantly in the lysosome (Fig. 1a) [23–25]. To explore the potential of shuttling intracellular tau to the proteasome for degradation, we engineered a chimeric anti-tau intrabody fused to ubiquitin harboring a K63R mutation favoring polyubiquitination at the K48 site. Conversely, to determine the efficiency of delivering intracellular tau for lysosomal-mediated degradation, we generated a second chimeric intrabody fused to ubiquitin harboring a K48R mutation favoring polyubiquitination at K63. For detection of our anti-tau intrabody, a C-terminal human influenza hemagglutinin (HA) tag was inserted. Next, we transfected HEK293t expressing h-tau with either a conventional anti-tau intrabody containing no distinct tags, or our modified proteasome targeting intrabody (intrabody^-K63R^), lysosomal targeting (intrabody^-K48R^) and control empty vector. At 72 h post-transfection, whole protein lysates were subjected to immunoblotting analysis for h-tau protein (anti-h-tau) and the intrabody (anti-HA). This revealed a decrease in h-tau protein levels upon the expression of both anti-tau intrabody^-K48R^ and intrabody^-K63R^ relative to the conventional unmodified anti-tau intrabody or empty vector control (Fig. 1b-c). The anti-tau intrabodies^-K48R^ and ^-K63R^ were much less abundant relative to the conventional anti-tau intrabody implicating these intrabodies are readily degraded together with h-tau.

To demonstrate the chimeric anti-tau intrabodies fused to ubiquitin harboring mutations at either K48 or K63 predominately target h-tau for proteasomal or lysosomal degradation, HEK293t cells co-expressing the various tau intrabodies with h-tau were treated with 50 μM MG132 (proteasome inhibitor) or 500 μM bafilomycin (lysosome inhibitor) (Fig. 1d). The decrease in h-tau protein levels upon expression of the anti-tau intrabody^-K48R^ was predominantly blocked by bafilomycin a classical lysosome inhibitor, with no effects on tau protein levels with inhibition of the proteasome by MG132 (Fig. 1d). In contrast, the decrease in h-tau protein levels upon expression of the anti-tau intrabody^-K63R^ was predominantly blocked by inhibition of the proteasome with MG132 and too a much lesser extent following lysosomal inhibition with bafilomycin (Fig. 1d). These analyses support anti-tau intrabodies harboring mutations at either K48 or K63 predominantly target h-tau for either proteasomal or lysosomal degradation.

We next determined if the chimerically fused intrabodies are polyubiqutinated by expressing the intrabodies in HEK293t cells followed by immunoprecipitation with an anti-HA antibody. The resultant intrabodies were then subjected to immunoblotting with anti-ubiquitin revealing that both chimerically fused intrabodies are polyubiquitinated (Additional file 1**:** Figure S1). Additionally, we determined if h-tau was polyubiquitinated following the expression of the chimeric intrabodies by immunoprecipitating h-tau from HEK293t cell lysates co-expressing the various anti-tau intrabodies or h-tau only. The resultant tau protein was then subjected to immunoblotting with anti-ubiquitin revealing little polyubiquitin following the co-expression of the chimerically fused intrabodies to ubiquitin. The small amount of polyubiquitin may potentially reflect the co-IP of the intrabodies which are polyubiquitinated (Additional file 1**:** Figure S1). These analyses suggest the chimerically fused anti-tau intrabodies are polyubiquitinated and shuttle h-tau for degradation.

### Expression of tau-degrading intrabodies in primary neurons expressing human tau protein reduce tau protein levels

**Fig. 2:**
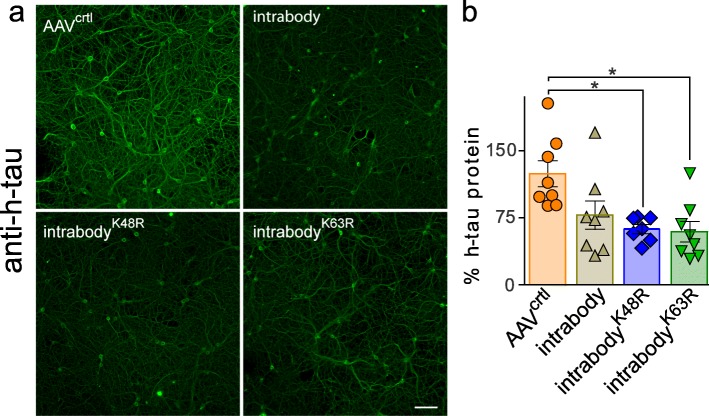
Expression of tau-degrading intrabodies decreases human tau in neurons. **a** Representative images of primary neurons co-expressing P301S-htau and the various anti-tau intrabodies. Expression of the conventional anti-tau intrabody, intrabody^-K48R^ or the intrabody^-K63R^ displayed a marked decrease in h-tau protein levels relative to the AAV-control; scale bar 50 μm. **b** P301S-htau protein quantification by tau ELISA in primary neurons co-expressing the various intrabodies revealed a significant decrease in h-tau protein levels upon expression of each tau-degrading intrabody relative to the AAV-controls. Expression of the conventional anti-tau intrabody displayed an non-significant decrease in h-tau protein relative to the AAV-control. Results are representative of three independent experiments, all data are expressed as mean ± s.e.m. and one-way ANOVA was performed with Tukey’s Multiple Comparison. *p < 0.05

The analyses in HEK293t cells demonstrated chimerically fused intrabodies with ubiquitin harboring lysine mutations that designate them for either proteasome or lysosomal-mediated degradation decreases intracellular h-tau protein levels. We next set out to determine the efficiency of degrading intracellular h-tau in neurons following AAV-mediated gene transfer of either the anti-tau intrabody^-K48R^, intrabody^-K63R^, or the conventional intrabody containing no targeting tags. In previous studies, we demonstrated that primary neuronal cultures transduced with AAVs encoding mutant P301S-htau for six hours before plating leads to stable expression of P301S-htau in neurons at 72 h post plating [26]. This paradigm enables the evaluation of the relative amount of P301S-htau protein following the co-expression of the anti-tau intrabodies and controls. Primary wild-type neurons expressing P301S-htau under a synapsin promoter were cultured for three days, followed by transduction with AAVs encoding each of the tau-degrading intrabodies or the conventional non-modified anti-tau intrabody driven by a chicken β-actin promoter. To assess the relative P301S-htau protein levels upon expression of the various intrabodies, the primary neurons were fixed at DIV10 for analysis by immunofluorescence (IF) (Fig. 2a). The IF analysis for P301S-htau protein revealed a decrease in P301S-htau protein levels throughout the dendrites, axons and cell bodies of neurons expressing either the conventional anti tau intrabody, anti-tau intrabody^K48R^, or intrabody^K63R^ relative to the AAV-control. To complement the h-tau IF analysis, we determined the amount of human P301S-htau protein from total neuronal lysates by ELISA. Wild type neurons expressing P301S h-tau were transduced with the various AAV-intrabodies and controls followed by harvesting neuronal lysates at DIV10 for ELISA analysis. ELISA measurements for h-tau protein from total lysates revealed a statistically significant decrease in P301S-htau protein levels following the expression of both the chimeric ubiquitin fused anti-tau intrabodies (K48R and K63R) relative to the control AAV. Analysis of the non-modified conventional intrabody revealed a non-significant trend toward lower tau protein levels relative to the control AAV (Fig. 2b). These analyses demonstrate that certain anti-tau intrabodies decrease intracellular tau protein levels in neurons.

### Expression of tau-degrading intrabodies in P301S-tg mice prior to overt tauopathy

**Fig. 3:**
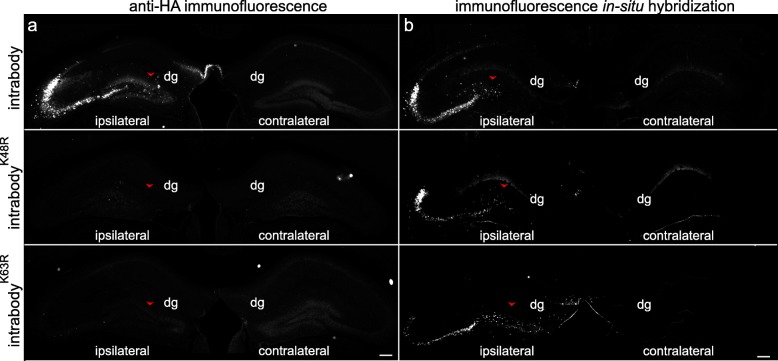
Immunofluorescence and fluorescence in situ analysis of anti-tau intrabody expression in aged P301S-tg mice. **a** Representative images of anti-HA for protein expression of the various anti-tau intrabodies in 9.5-month-old P301S-tg mice. Immunofluorescent detection of HA reveals clear-cut evidence of the non-targeting anti-tau intrabody within the ipsilateral hippocampus, whereas there was little to no immunofluorescence signal with the modified anti-tau intrabodies. Arrowhead indicating the injection site within the dentate gyrus (dg). **b** Representative images of in situ hybridization in 9.5-month-old P301S-tg mice validated the expression of the mRNA of the various anti-tau intrabodies within the hippocampus ipsilateral to the AAV injection. Scale bar 200 μM

We have hypothesized the expression of anti-tau intrabodies in neurons will decrease intracellular tau protein levels leading to a greater effect relative to conventional immunotherapeutic approaches for tauopathies. Therefore, following the validation that certain anti-tau intrabodies significantly reduced intracellular h-tau protein levels in primary neurons, we set to evaluate the efficacy of the anti-tau intrabodies on delaying or decreasing tauopathy in aged P301S-tg mice. P301S-tg mice develop age-dependent tauopathy that begins at ~ 5–6 months of age displaying moderate pathological tau accumulation in the hippocampus as assessed with an anti-phosphorylated tau antibody AT8 (Additional file 1: Figure S2). AT8 targets phosphorylated tau (p-tau) at residues Ser202 and Thr205 that is present in aggregated forms of tau, including paired helical filaments and in other pathological conformations. To determine the effects of expressing the different anti-tau intrabodies prior to overt tauopathy, we unilaterally injected AAVs encoding either the chimeric anti-tau intrabodies fused to ubiquitin (intrabody^-K48R^, intrabody^-K63^), conventional intrabody, or AAV-control into the 6 month old P301S-tg mice (*n* = 4–7 per condition). This time point is consistent with early disease displaying a minimal deposition of p-tau in the hippocampus (Additional file 1: Figure S1). Following 12 weeks post-injection, the mice were euthanized for histopathological analysis. To validate the expression of each anti-tau intrabody we subjected the cohort of 9.5 month old P301S-tg mice to immunofluorescence analysis with anti-HA. Although, anti-HA staining revealed the presence of the conventional anti-tau intrabody protein containing no targeting tags throughout the dentate gyrus mossy fiber regions of the hippocampus, detection of either the intrabody^-K48R^ or intrabody^-K63R^ were much less evident (Fig. 3a). The low protein detection of the tau-degrading intrabodies parallels our initial characterization in cell culture analysis suggesting a rapid degradation of these anti-tau intrabodies in vivo (Fig. 1**)**. Therefore, to validate the expression of each anti-tau intrabody, we performed fluorescence in situ hybridization (FiSH) utilizing a probe that extended the length of the variable regions of the heavy and light chains of the anti-tau intrabody (Fig. 3b). This analysis revealed expression of each anti-tau intrabody unilaterally in the dentate gyrus, CA2, and CA3 regions but not in the CA1 cell layer of the hippocampus of 9.5 month old P301S-tg mice.

### Expression of tau-degrading intrabodies prior to overt tau pathology prevented tauopathy in aged P301S-tg mice

**Fig. 4:**
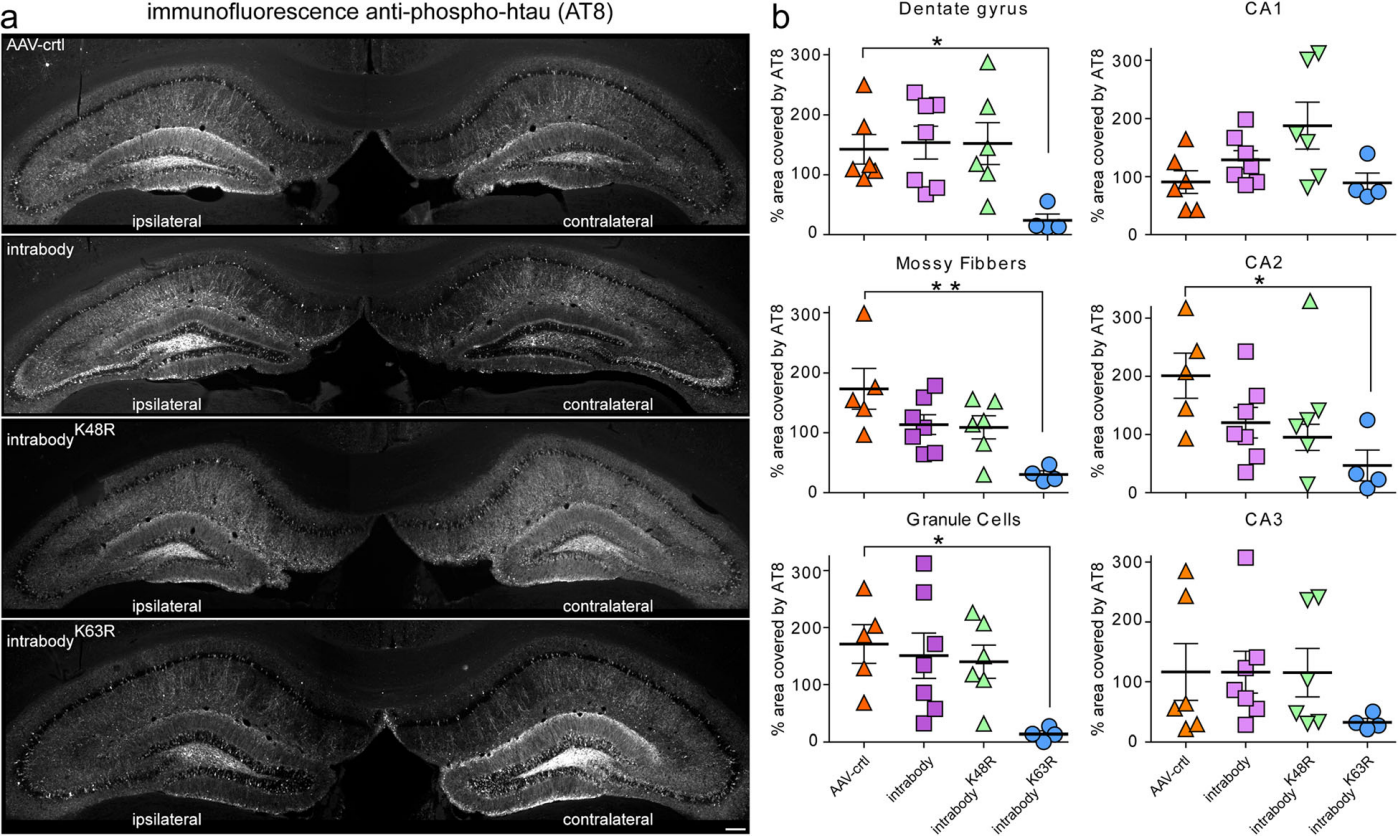
Tau-degrading intrabodies prevent tauopathy at early-disease in P301S-tg mice. **a** Representative images of pathological tau accumulation as seen with AT8 staining in 9.5 month-old P301S-tg mice following the expression of anti-tau intrabodies prior to overt tau pathology (early-disease). **b** AAV-control displayed an increase in pathological tau accumulation in the ipsilateral hippocampal side**.** Expression of the conventional anti-tau intrabody displayed a non-significant increase in pathological tau accumulation in the dentate gyrus, granule cells, mossy fibers and CA2 region of the ipsilateral relative to the contralateral hippocampus. Expression of the chimeric tau-degrading intrabody fused to ubiquitin harboring a K48R mutation prone for lysosomal-meditated degradation displayed a non-significant increase in pathological tau accumulation in the granule cells, CA1 and CA3 region of the ipsilateral relative to the contralateral hippocampus. Expression of the chimeric tau-degrading intrabody fused to ubiquitin harboring a K63R mutation prone for proteasome-mediated degradation displayed a significant decrease in pathological tau accumulation in the ipsilateral hippocampal dentate gyrus, granule cells and mossy fibers ipsilateral relative to the contralateral hippocampus. Scale bar 200 μM. Quantification (*n* = 4–7) of pathological tau from the ipsilateral relative to the contralateral dentate gyrus. All data are mean ± s.e.m. one-way ANOVA with Tukey’s Multiple Comparison. *p < 0.05, **p < 0.01

Upon validating the expression for each intrabody unilaterally in the hippocampus of aged P301S-tg mice, we next assessed the efficiency for the tau-degrading intrabodies to prevent or delay the accumulation of pathological tau. At 9.5 months old, P301S-tg mice displayed a substantial amount of p-tau in the hippocampus (Additional file 1: Figure S2) To determine the percent decrease of pathological tau following the expression of the various anti-tau intrabodies at early-disease, an average ratio of p-tau positive staining from the contralateral (non-injected) side to the ipsilateral (injected region) was measured within the dentate gyrus, granule cells, mossy fibers, CA1, CA2, and CA3 regions of the hippocampus. This analysis revealed a statistically significant decrease in pathological tau within the ipsilateral CA2, dentate gyrus, mossy fibers, and the granule cell layer expressing the chimeric tau-degrading intrabody fused to ubiquitin harboring a K63R mutation relative to the control AAV (Fig. 4a-b). In contrast, measurements for the accumulation of pathological tau in the CA1 hippocampal cell layer that lack expression of the intrabodies indicated a comparable percent area covered by tau pathology within the ipsilateral and contralateral hippocampus for all intrabodies (Fig. 4a-b). Intriguingly, the expression of either the conventional anti-tau intrabody or the intrabody fused to ubiquitin harboring a K48R mutation was ineffective in delaying or eliminating tauopathy in in vivo which contrasted with our data in HEK293t cells and primary neuronal cultures (Figs. 1 and 2).

In additional studies, we determined the total tau protein levels following the expression of the various anti-tau intrabodies at early-disease, an average ratio of total human tau positive staining (anti-HT7) from the contralateral (non-injected) side to the ipsilateral (injected region) was measured within the dentate gyrus, granule cells, mossy fibers, CA1, CA2, and CA3 regions of the hippocampus. This analysis revealed a statistically significant decrease in h-tau within the ipsilateral CA2 following the expression of both the chimeric tau-degrading intrabody fused to ubiquitin harboring a K63R mutation. Additionally, there was a non-significant trend toward lowering of h-tau levels within the CA1, CA3, mossy fibers and dentate gyrus following the expression of the anti-tau intrabody harboring a K63R mutation (Additional file 1: Figure S3).

Collectively, these analyses suggest intrabodies that predominantly target tau for proteasomal degradation are more proficient in degrading pathological tau relative to conventional intrabodies in vivo in human tau transgenic mice.

### Expression of tau-degrading intrabodies reduce tauopathy in aged P301S-tg mice

We next assessed the efficiency for our tau-degrading intrabodies to eliminate or decrease tau pathology after extensive tauopathy is already deposited in the hippocampus of P301S-tg mice (mid-disease). At 8.5 months P301S-tg mice display substantial deposition of pathological tau throughout the hippocampus predominantly in the dentate gyrus mossy fibers (Additional file 1: Figure S2). Therefore, we unilaterally injected AAVs encoding either anti-tau intrabody^-K48R^, intrabody^-K63R^, conventional intrabody, or AAV-control into the dentate gyrus at 8.5 months of P301S-tg mice (*n* = 6–8 per condition). Six weeks post-injection, the mice were euthanized for histopathological analysis to assess the expression of each anti-tau intrabody and the effects on tau pathology.

Similar to what we observed with injections at 6 months of age, prior to overt tau pathology (Fig. 3), anti-HA staining revealed protein expression for the conventional anti-tau intrabody containing no targeting tags throughout the dentate gyrus-mossy fibers of the hippocampus (Additional file 1: Figure S4a). Whereas, anti-HA staining of either the intrabody^-K48R^ or intrabody^-K63R^ were much less evident (Additional file 1: Figure S4a). Nonetheless, FiSH analysis validated the expression of each anti-tau intrabody unilaterally in the dentate gyrus, CA2, and CA3 regions but not in the CA1 cell layer of the hippocampus of P301S-tg mice (Additional file 1: Figure S4b).

**Fig. 5:**
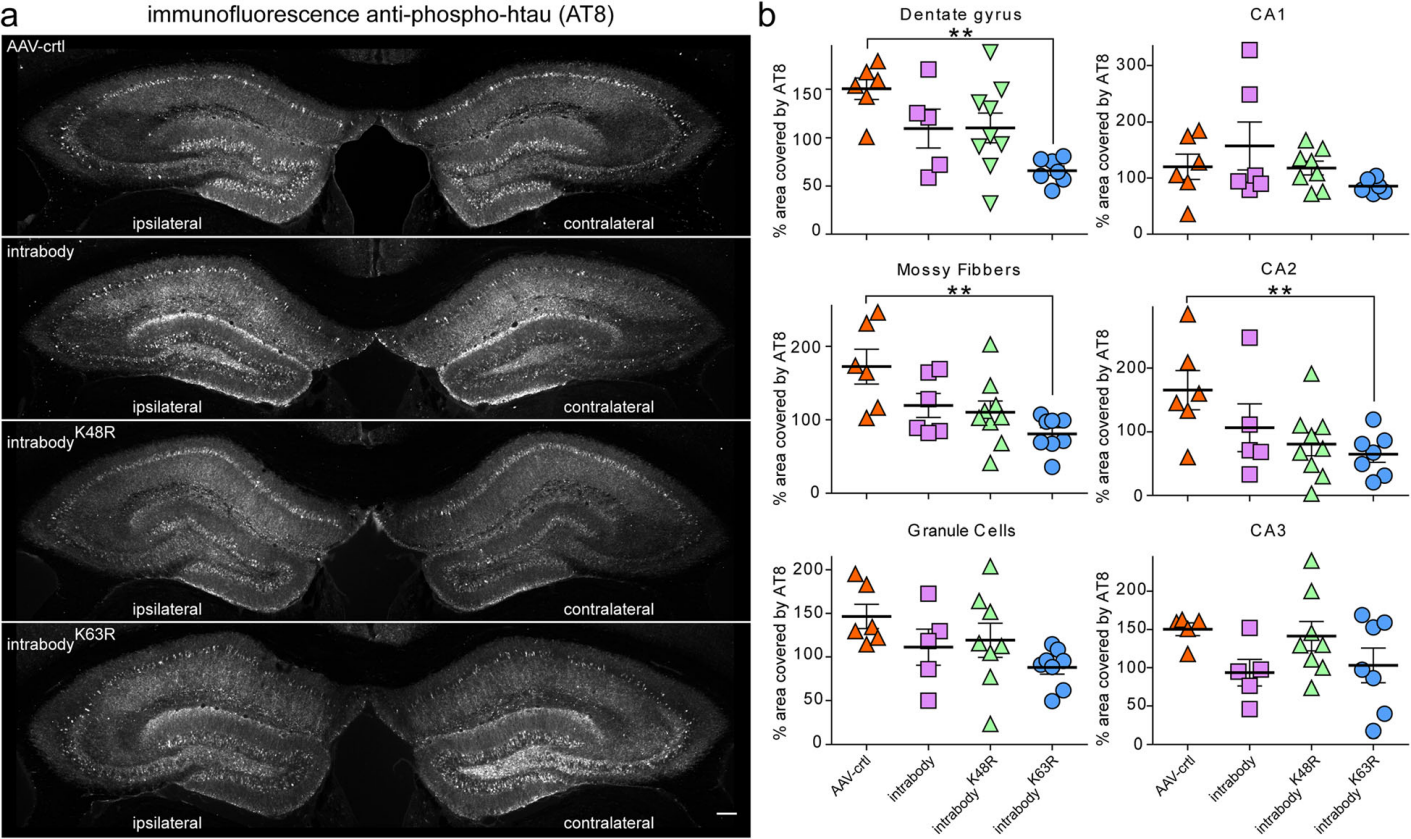
Tau-degrading intrabodies eliminate tauopathy at mid-disease in P301S-tg mice. **a.** Representative images of pathological tau accumulation as seen with AT8 staining in 9.5 month-old P301S-tg mice following the expression of anti-tau intrabodies after tauopathy deposition (mid-disease). **b** AAV-control displayed an increase in pathological tau accumulation in the ipsilateral hippocampal side**.** Expression of the conventional anti-tau intrabody displayed a non-significant increase in pathological tau accumulation in the dentate gyrus, granule cells, mossy fibers and CA2 ipsilateral relative to the contralateral hippocampus. Expression of the chimeric tau-degrading intrabody fused to ubiquitin harboring a K48R mutation prone for lysosomal-meditated degradation displayed a non-significant increase in pathological tau accumulation in the granule cells, CA1 and CA3 regions of the ipsilateral relative to the contralateral hippocapus. Expression of the chimeric tau-degrading intrabody fused to ubiquitin harboring a K63R mutation prone for proteasome-mediated degradation displayed a significant decrease in pathological tau accumulation in the hippocampal dentate gyrus, granule cells and mossy fibers in the ipsilateral relative to the contralateral hippocampus. Scale bar 200 μM. Quantification (*n* = 6–9) of pathological tau from the contralateral to the ipsilateral dentate gyrus. All data are mean ± s.e.m. one-way ANOVA with Tukey’s Multiple Comparison. *p < 0.05, **p < 0.01

Following the validating for the expression for each intrabody unilaterally in the hippocampus of aged P301S-tg mice, we next assessed the efficiency for the tau-degrading intrabodies to eliminate or decrease related tauopathy. Two hippocampal sections from each P301S-tg mouse expressing the various intrabodies were subjected to immunohistological analysis for p-tau (AT8) and quantified for the percent decrease of pathological tau within the dentate gyrus, granule cells, mossy fibers, CA1, CA2, and CA3 regions of the hippocampus as previously. This analysis revealed a statistically significant decrease in pathological tau within the ipsilateral dentate gyrus and mossy fibers expressing the chimeric tau-degrading intrabody fused to ubiquitin harboring a K63R mutation relative to the AAV-control (Fig. 5a-b). Measurements for the accumulation of pathological tau in the CA1 hippocampal cell layer that lack expression of the tau intrabodies indicated a comparable percent area covered by tau pathology within the ipsilateral and contralateral hippocampus (Fig. 5a-b). In line with our analyses at an early-disease state when treatment was started (Fig. 4), the expression of the conventional anti-tau intrabody and the intrabody^-K48R^ at a mid-disease stage were ineffective in eliminating or decreasing tauopathy in vivo in P301S-tg mice (Fig. 5a-b). Collectively, these analyses further validate tau-degrading intrabodies destined for proteasomal mediated degradation are more proficient in degrading pathological tau relative to a conventional intrabody in P30S-tg mice.

## Discussion

By utilizing ubiquitin, we have engineered anti-tau intrabodies that are prone to target tau for either the proteasome or lysosomal-degradation to identify the most proficient mechanism for reducing tauopathy. We demonstrate an anti-tau intrabody chimerically fused to ubiquitin harboring a mutation prone for proteasome degradation significantly reduced tauopathy in aged P301S-tg mice prior to overt tauopathy (early disease) and after substantial pathological tau deposition (late disease) relative to the conventional intrabody and controls. Follow up studies should aim at evaluating the global expression of the tau-degrading intrabodies and their efficacy in preventing brain atrophy and behavioral abnormalities of aged P301S-tg mice.

Intriguingly, despite all three intrabodies displaying the ability to reduce tau in HEK293t cells and primary neuronal cultures, the chimeric intrabody fused to ubiquitin harboring a mutation prone for lysosome-mediated degradation failed to prevent tauopathy in aged P301S mice after the onset of disease. One possibility is that the decreased efficacy seen with the K48R mutation may stem from the fact that polyubiquitination at K63 has been shown to display a variety of cellular functions in addition to lysosomal degradation that includes DNA repair and internalization of plasma membrane proteins [27–29]. Thus, the ubiquitin harboring a K48R mutation in vivo may be less prone for lysosomal degradation or perhaps the pathophysiological events leading to tauopathy decreases the efficiency of this particular intrabody’s ability to allow for targeted lysosomal degradation [30, 31]. Alternatively, the anti-tau intrabody^-K48R^ is rapidly degraded before binding to tau protein leading to a decrease in efficacy. Future studies should aim at evaluating whether targeting other tau mutants for proteasomal degradation also display a higher efficacy relative to the lysosomal degradation with our tau degrading intrabodies.

Bi-functional intrabodies directed toward the proteasome targeting other proteinopathies (e.g. Huntingtin and TAR DNA-binding protein 43) have been proposed in previous studies by fusing a PEST domain to certain scFv [32, 33]. Proteins with PEST domains display a short half-life, thus intrabodies fused to a PEST domain mimic a short half-life and are rapidly degraded. Although, conceptually PEST-intrabodies decreased the targeted pathological protein in these prior studies, the effects were demonstrated only in cellular culture models by the co-expression of the intrabody with the pathological protein [32, 33]. In the current study, we evaluated a disconnect between the ability for a chimeric intrabody fused to ubiquitin harboring a mutation prone for lysosome-mediate degradation to reduce tau protein levels in primary neuronal cultures/HEK293t cells and its inability to reduce tauopathy in vivo in aged P301S-tg mice. This disconnect illustrates that although cell culture models are important for rational design, the complexity of in vivo models do not always translate in vivo. Therefore, the therapeutic potential or the extent to which PEST-intrabodies prevent proteinopathies in vivo in aging mammalian models of proteinopathies remains largely unknown. Moreover, the efficiency for PEST-intrabodies to reduce the proteinopathy after the onset of disease also remains a critical question that is unanswered and is important for a thorough evaluation.

## Conclusions

Compelling evidence from several studies has demonstrated immunotherapies targeting h-tau are neuroprotective in mouse models of tauopathies [10–17]. However, conventional immunotherapeutic approaches have some limitations that may preclude their full potential when targeting tauopathy. Particularly, immunotherapy by most approaches is limited to targeting extracellular pathological proteins, whereas the majority of pathological tau remains intracellular in the cytosol where it is not likely to be able to interact with anti-tau mAB or secreted scFvs. In the current study, we describe a new immunotherapeutic approach that demonstrates potential for overcoming this limitation by targeting the degradation of h-tau intracellularly in the cytosol with tau-degrading intrabodies following AAV-mediated gene transfer into the brain. Combining this approach with the recent advances of AAV-mediated gene transfer that provides global-neuronal transduction in the adult mouse CNS by intravenous administration may provide a feasible approach for potential long-term treatment of tauopathies.

## Supplementary information

**Additional file 1: Figure S1**. anti-tau intrabodies fused to ubiquitin harboring a K48 or K63 mutation are polyubiquitinated. **Figure S2**. P301S-tg mice display aged dependent pathological tau accumulation. **Figure S3**. Tau-degrading intrabodies decrease tau protein levels in P301S-tg mice. **Figure S4**. Expression of anti-tau intrabodies in aged P301S-tg mice analyzed by immunofluorescence and fluorescence in situ analysis.

## Data Availability

All raw data used and analyzed for the current study are available from the corresponding author on reasonable request.

## Abbreviations

CNS: Central nervous system
FiSH: Fluorescence in situ hybridization
HA: Influenza hemagglutinin
h-tau: Human tau
mABs: Monoclonal antibodies
MBD: Microtubule-binding domain
P301S-tg: Transgenic mice expressing human P301S tau
scFv: Single-chain variable fragment

## References

[1] Joachim CL (1987). Tau antisera recognize neurofibrillary tangles in a range of neurodegenerative disorders. Ann Neurol.

[2] Lee VM, Goedert M, Trojanowski JQ (2001). Neurodegenerative tauopathies. Annu Rev Neurosci.

[3] Weingarten MD (1975). A protein factor essential for microtubule assembly. Proc Natl Acad Sci U S A.

[4] Witman GB (1976). Tubulin requires tau for growth onto microtubule initiating sites. Proc Natl Acad Sci U S A.

[5] Trojanowski JQ (1989). Distribution of tau proteins in the normal human central and peripheral nervous system. J Histochem Cytochem.

[6] Zempel H, Mandelkow E. Lost after translation: missorting of tau protein and consequences for Alzheimer disease. Trends Neurosci. 2014. 10.1016/j.tins.2014.08.004 25223701

[7] Clavaguera F (2009). Transmission and spreading of tauopathy in transgenic mouse brain. Nat Cell Biol.

[8] Lasagna-Reeves CA (2012). Alzheimer brain-derived tau oligomers propagate pathology from endogenous tau. Sci Rep.

[9] Iba M (2013). Synthetic tau fibrils mediate transmission of neurofibrillary tangles in a transgenic mouse model of Alzheimer's-like tauopathy. J Neurosci.

[10] Bi M (2011). Tau-targeted immunization impedes progression of neurofibrillary histopathology in aged P301L tau transgenic mice. PLoS One.

[11] Boimel M (2010). Efficacy and safety of immunization with phosphorylated tau against neurofibrillary tangles in mice. Exp Neurol.

[12] Asuni AA (2007). Immunotherapy targeting pathological tau conformers in a tangle mouse model reduces brain pathology with associated functional improvements. J Neurosci.

[13] Boutajangout A, Quartermain D, Sigurdsson EM (2010). Immunotherapy targeting pathological tau prevents cognitive decline in a new tangle mouse model. J Neurosci.

[14] Dai CL (2018). Tau passive immunization blocks seeding and spread of Alzheimer hyperphosphorylated tau-induced pathology in 3 x Tg-AD mice. Alzheimers Res Ther.

[15] Liu W (2016). Vectored Intracerebral immunization with the anti-tau monoclonal antibody PHF1 markedly reduces tau pathology in mutant tau transgenic mice. J Neurosci.

[16] Chai X (2011). Passive immunization with anti-tau antibodies in two transgenic models: reduction of tau pathology and delay of disease progression. J Biol Chem.

[17] Yanamandra K (2013). Anti-tau antibodies that block tau aggregate seeding in vitro markedly decrease pathology and improve cognition in vivo. Neuron.

[18] Yanamandra K (2015). Anti-tau antibody reduces insoluble tau and decreases brain atrophy. Ann Clin Transl Neurol.

[19] Yoshiyama Y (2007). Synapse loss and microglial activation precede tangles in a P301S tauopathy mouse model. Neuron.

[20] Ising C (2017). AAV-mediated expression of anti-tau scFvs decreases tau accumulation in a mouse model of tauopathy. J Exp Med.

[21] Nicoll JA (2003). Neuropathology of human Alzheimer disease after immunization with amyloid-beta peptide: a case report. Nat Med.

[22] Chau V (1989). A multiubiquitin chain is confined to specific lysine in a targeted short-lived protein. Science.

[23] Ferreira JV (2015). K63 linked ubiquitin chain formation is a signal for HIF1A degradation by chaperone-mediated autophagy. Sci Rep.

[24] Olzmann JA, Chin LS (2008). Parkin-mediated K63-linked polyubiquitination: a signal for targeting misfolded proteins to the aggresome-autophagy pathway. Autophagy.

[25] Tan JM (2008). Lysine 63-linked ubiquitination promotes the formation and autophagic clearance of protein inclusions associated with neurodegenerative diseases. Hum Mol Genet.

[26] Shi Y (2017). ApoE4 markedly exacerbates tau-mediated neurodegeneration in a mouse model of tauopathy. Nature.

[27] Duncan LM (2006). Lysine-63-linked ubiquitination is required for endolysosomal degradation of class I molecules. EMBO J.

[28] Galan JM, Haguenauer-Tsapis R (1997). Ubiquitin lys63 is involved in ubiquitination of a yeast plasma membrane protein. EMBO J.

[29] Geetha T, Jiang J, Wooten MW (2005). Lysine 63 polyubiquitination of the nerve growth factor receptor TrkA directs internalization and signaling. Mol Cell.

[30] Bahr BA, Bendiske J (2002). The neuropathogenic contributions of lysosomal dysfunction. J Neurochem.

[31] Nixon RA, Cataldo AM (2006). Lysosomal system pathways: genes to neurodegeneration in Alzheimer's disease. J Alzheimers Dis.

[32] Butler DC, Messer A (2011). Bifunctional anti-huntingtin proteasome-directed intrabodies mediate efficient degradation of mutant huntingtin exon 1 protein fragments. PLoS One.

[33] Tamaki Y (2018). Elimination of TDP-43 inclusions linked to amyotrophic lateral sclerosis by a misfolding-specific intrabody with dual proteolytic signals. Sci Rep.

