# Supplementary material for: Targeting tauopathy with engineered tau-degrading intrabodies
Source: Mol Neurodegener. 2019 Oct 22;14:38. doi: 10.1186/s13024-019-0340-6 (PMC6805661; doi:10.1186/s13024-019-0340-6)
Supplement: Supplementary file 1 — Additional file 1: Figure S1. anti-tau intrabodies fused to ubiquitin harboring a K48 or K63 mutation are polyubiquitinated. Figure S2. P301S-tg mice display aged dependent pathological tau accumulation. Figure S3. Tau-degrading intrabodies decrease tau protein levels in P301S-tg mice. Figure S4. Expression of anti-tau intrabodies in aged P301S-tg mice analyzed by immunofluorescence and fluorescence in situ analysis. [file 13024_2019_340_MOESM1_ESM.docx]

**
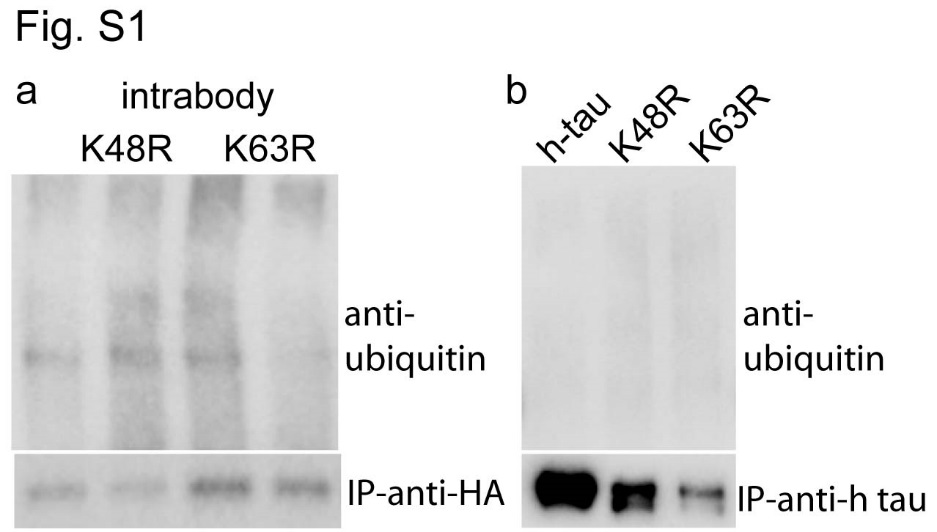
**

**Figure S1. anti-tau intrabodies fused to ubiquitin harboring a K48 or K63 mutation are polyubiquitinated.**

**a** Immunoprecipitation of anti-tau intrabodies from HEK293t cell lysates were subjected immunoblotting analysis with anti-ubiquitin. The immunoprecipitated anti-tau intrabodies revealed polyubiquitination. **b** Immunoprecipitated h-tau from HEK293t cell lysate co-expressing the anti-tau intrabody^K48R^ or intrabody^K63R^ were subjected to immunoblotting analysis with anti-ubiquitin revealed a little polyubiquitination.


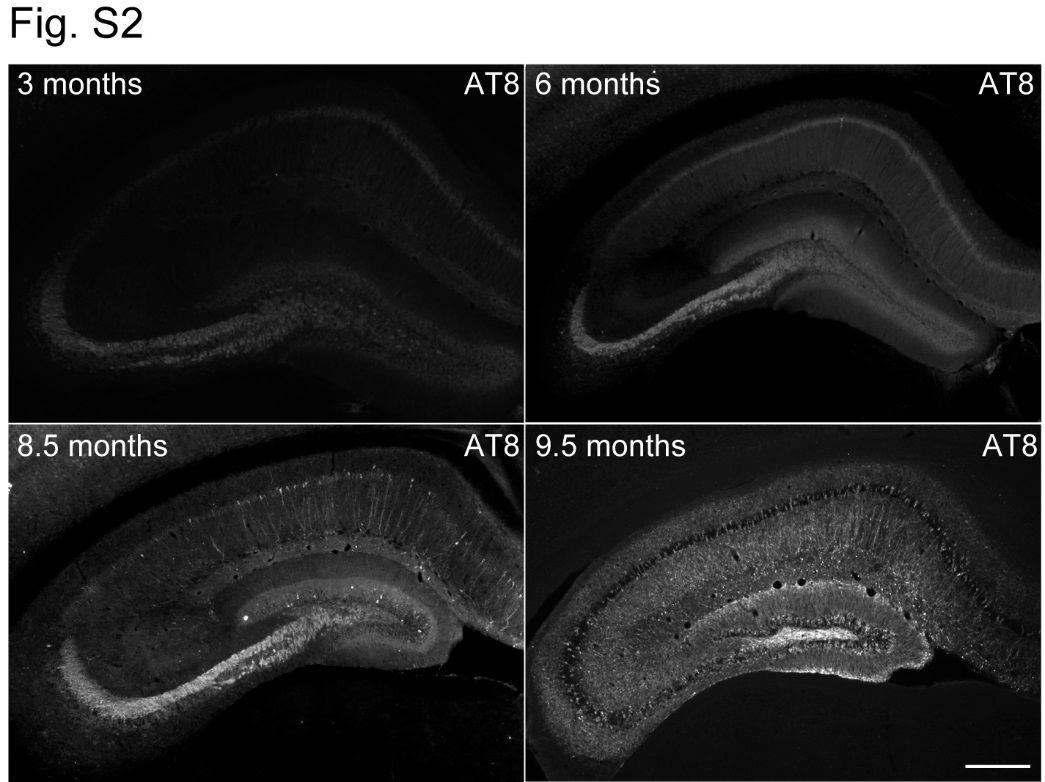


**Figure S2. P301S-tg mice display aged dependent pathological tau accumulation.**

Representative images of anti-AT8 staining from 3, 6, 8.5 and 9.5 month old P301S-tg mice demonstrating an aged dependent increase in the accumulation of pathological tau. Scale bar 200μm

**
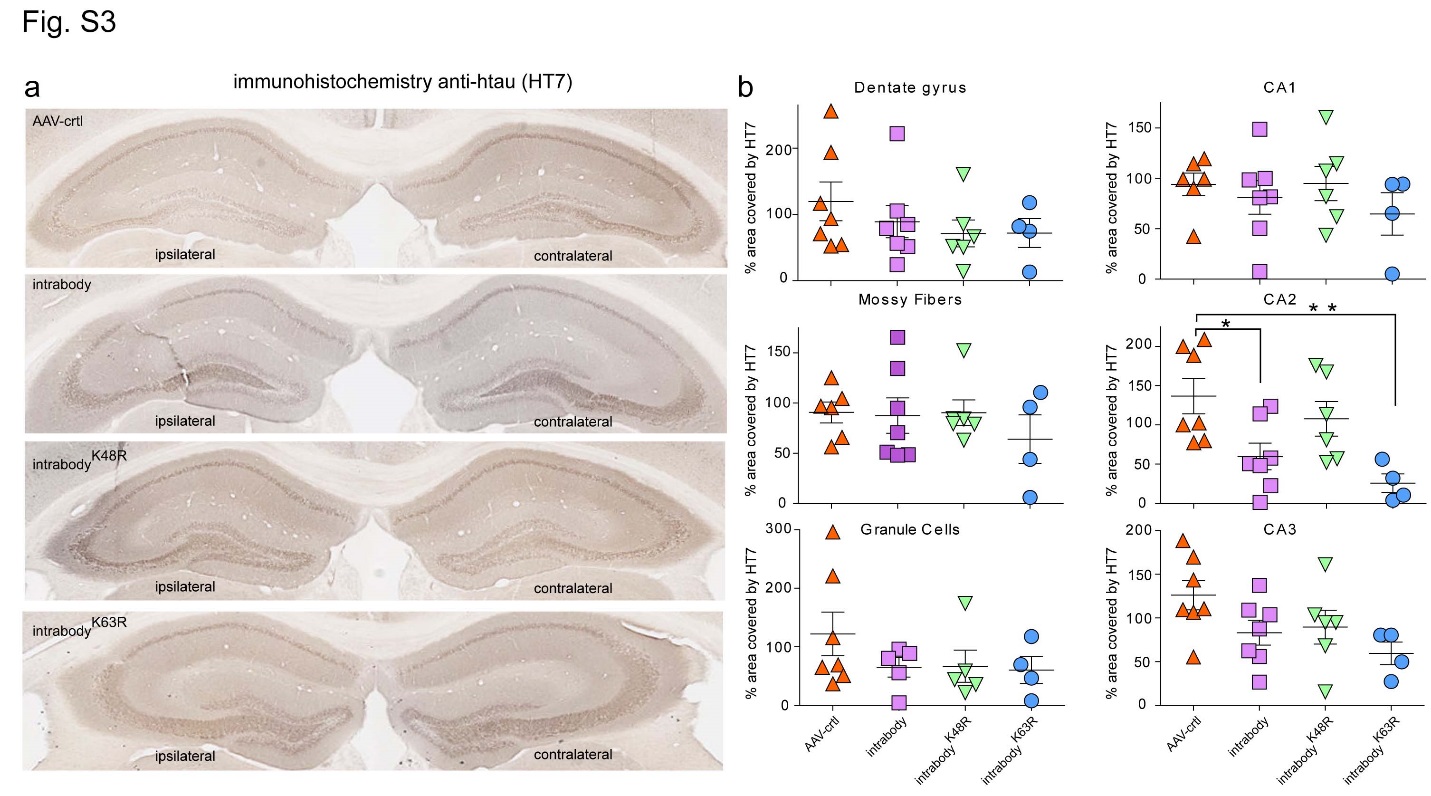
Figure S3. Tau-degrading intrabodies decrease tau protein levels in P301S-tg mice**

**a** Representative images of total tau staining in 9.5 month old P301S-tg mice following the expression of anti-tau intrabodies prior to overt tau pathology (early-disease). **b** Expression of the chimeric tau-degrading intrabody fused to ubiquitin harboring a K63R mutation prone for proteasome-mediated degradation displayed a significant decrease in human tau in the ipsilateral hippocampal CA2 region and non-significant trend toward lower tau levels within the CA1, CA3, mossy fibers and dentate gyrus. All data are mean ± s.e.m. one-way ANOVA with Tukey’s Multiple Comparison. *p<0.05, **p<0.01.

**
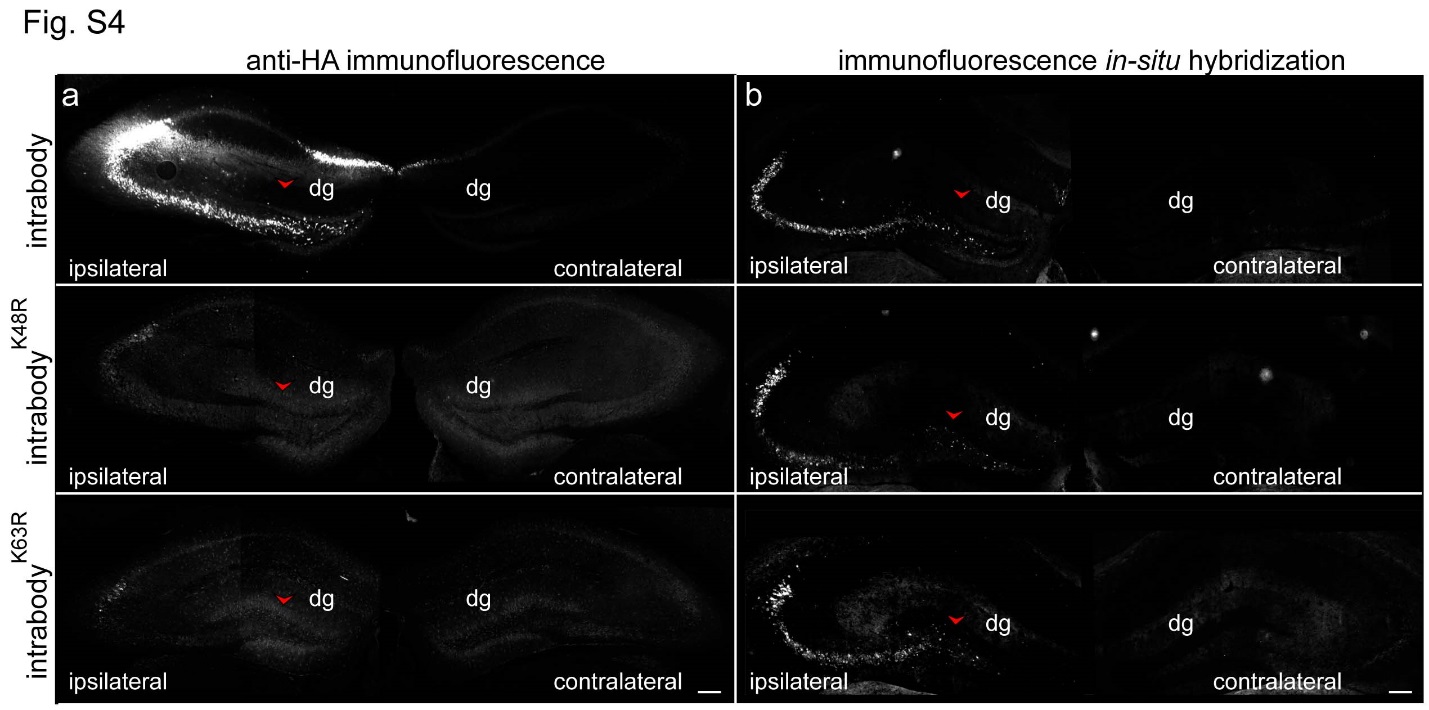
**

**Figure S4. Expression of anti-tau intrabodies in aged P301S-tg mice analyzed by immunofluorescence and fluorescence *in situ* analysis.**

**a** Representative images of anti-HA for protein expression of the various anti-tau intrabodies in 9.5-month-old P301S-tg mice revealing the expression of the conventional anti-tau intrabody within the ipsilateral hippocampus, whereas there was little to no immunofluorescence signal with the modified anti-tau intrabodies. Arrowhead indicating the injection site within the Dentate Gyrus. **b** Representative images of *in situ* hybridization in 9.5-month-old P301S-tg mice validated the expression of the mRNA of the various anti-tau intrabodies within the hippocampus ipsilateral to the AAV injection. Scale bar 200μM
